# Mechanism of saikogenin G against major depressive disorder determined by network pharmacology

**DOI:** 10.1097/MD.0000000000030193

**Published:** 2022-08-26

**Authors:** Lili Hu, Jue Wang, Xiaoge Zhao, Donghui Cai

**Affiliations:** a College of Basic Medicine, Shanxi University of Chinese Medicine, Jinzhong, China; b Key Laboratory of Environment and Genes Related to Diseases, Ministry of Education of China, Xi’an Jiaotong University, Xi’an, China.

**Keywords:** major depressive disorder, network pharmacology, saikogenin G

## Abstract

Many classic decoctions of Chinese medicine including Radix Bupleuri are used to treat major depressive disorder (MDD). Saikosaponin D is a representative bioactive ingredient discovered in Radix Bupleuri. The mechanism of saikogenin G (SGG) as a metabolite in MDD remains unclear to date. This study aims to elucidate the mechanism of SGG in treating MDD with network pharmacology.

We evaluated the drug likeness of SGG with SwissADME web tool and predicted its targets using the SwissTargetPrediction and PharmMapper. MDD-related targets were identified from the following databases: DisGeNET, DrugBank, Online Mendelian Inheritance in Man, and GeneCards. The common targets of SGG and MDD were imported to the STRING11.0 database, and then a protein–protein interaction network was constructed. Gene ontology and Kyoto Encyclopedia of Genes and Genomes pathway enrichment were analyzed with DAVID 6.8 database.

The molecular weight of SGG was 472.7 g/mol, the topological polar surface area was 69.92 A^2^ <140 A^2^, the octanol/water partition coefficient (Consensus LogP_0/W_) was 4.80, the rotatable bond was 1, the hydrogen bond donors was 3, and the hydrogen bond acceptors was 4. A total of 322 targets of SGG were obtained and there were 1724 MDD-related targets. A total of 78 overlapping genes were selected as targets of MDD treatment including albumin, insulin-like growth factor I, mitogen-activated protein kinase 1, proto-oncogene tyrosine-protein kinase Src, and epidermal growth factor receptor. Gene ontology and Kyoto Encyclopedia of Genes and Genomes enrichment analysis suggested that proteoglycans in cancer, pathways in cancer, prostate cancer, hypoxia-inducible factor-1, central carbon metabolism in cancer, estrogen, PI3K-Akt, ErbB, Rap1, and prolactin signaling pathways played an important role(*P* < .0001).

This study showed that SGG exhibits good drug-like properties and elucidated the potential mechanisms of SGG in treating MDD with regulating inflammation, energy metabolism, monoamine neurotransmitters, neuroplasticity, phosphocreatine-creatine kinase circuits, and so on.

## 1. Introduction

Depressive disorder is a common mental disorder. Main subcategories of depressive disorders include major depressive disorder (MDD) and dysthymia.^[1]^ About 322 million people in the world are suffering from depression. Recurrent and long-lasting depression is characterized by continuous sadness, lack of interest, sense of guilt, poor attention, and so on. People with depression cannot work and live normally, commit nonsuicidal self-injury, or even suicide in serious cases.^[2]^ Between 50% and 70% of suicide victims, which was 788,000 in 2015, may be affected by MDD.^[1,3]^

Many classic decoctions of Chinese medicine are used to treat MDD. Chaihu Shugan Powder (*Citrus reticulata*, Radix Bupleuri, Chuanxiong Rhizoma, Cyperi Rhizoma, Aurantii Fructus, Paeoniae Radix Alba, licorice), Xiaoyao Powder (Radix Bupleuri, *Atractylodes macrocephala* Koidz, Paeoniae Radix Alba, Menthae Herba, Angelicae Sinensis Radix, *Poria cocos* (Schw.) Wolf., licorice, *Zingiber officinale* Roscoe), Sini Power (Radix Bupleuri, Paeoniae Radix Alba, Aurantii Fructus Immaturus, licorice), and Xiaochaihu decoction (Radix Bupleuri, *Arum ternatum Thunb., Panax Ginseng* C. A. Mey., licorice, Scutellariae radix, *Z. officinale* Roscoe, Jujubae Fructus) have been used for over thousands of years in China to treat MDD.^[4]^

Saikosaponins (Saikosaponin A, Saikosaponin B, Saikosaponin D, and Saikosaponin K) are representative bioactive ingredients discovered in Radix Bupleuri, a common component of the mentioned decoctions above. Saikosaponin D protects the nervous system, alleviates neurological dysfunction, rescues sexual behavior deficits, improves depression-like behavior, and prevents Alzheimer disease, osteoarthritis, breast cancer, and leukemia.^[5–11]^ Saikosaponin D as a triterpene saponin glycoside is hydrolyzed to prosaikogenin G and then to saikogenin G (SGG), which is subsequently absorbed into the circulatory system.^[12]^ Previous studies focused on the biological effects of saikosaponin D. However, the bioactivity and mechanism of SGG in MDD remain unclear to date.

In the present study, we aimed to elucidate the mechanism of SGG in treating MDD with network pharmacology. We evaluated the druglikeness of SGG and predicted its targets against MDD. A protein–protein interaction (PPI) network was constructed with the common targets. Gene ontology (GO) and Kyoto Encyclopedia of Genes and Genomes (KEGG) pathway enrichment were analyzed with the database. This study provided a thorough investigation and comprehensive analysis of SGG in treating MDD.

## 2. Methods

### 2.1. Chemical structure and druglikeness analysis of SGG

The molecular structure file and canonical SMILES of SGG were obtained from the PubChem database (https://pubchem.ncbi.nlm.nih.gov/).^[13]^ Lipinski rule of 5 (RO5) was evaluated by the SwissADME web tool (http://www.swissadme.ch/). The topological polar surface area, LogS, gastrointestinal absorption, blood–brain barrier, Log Kp (skin permeation), and druglikeness were also analyzed.

### 2.2. Target identification of SGG in MDD

The SwissTargetPrediction (http://www.swisstargetprediction.ch/index.php) and PharmMapper (http://www.lilab-ecust.cn/pharmmapper/submitfile.html) databases were conducted to predict the targets of SGG.^[14,15]^

A comprehensive collection of disease-related genes were obtained from the DisGeNET (https://www.disgenet.org/search),^[16]^ DrugBank (https://go.drugbank.com/),^[17]^ Online Mendelian Inheritance in Man (http://www.omim.org),^[18]^ and GeneCards (https://www.genecards.org/) databases by using “major depressive disorder” as a key phrase for MMD. The SGG- and MDD-related targets were sent to the UniProt database (http://www.uniprot.org/) for normalization. The intersection of identified candidate targets was evaluated by Venny 2.1 (https://bioinfogp.cnb.csic.es/tools/venny/index.html). The shared targets represent the targets of SGG against MDD.

### 2.3. PPI network analysis

A PPI network was predicted based on the STRING 11.0 platform (https://string-db.org/). The targets of SGG against MDD were imported to the STRING11.0 database, and then target interaction information was combined and analyzed with Cytoscape software (version 3.7.1; https://www.cytoscape.org/).

### 2.4. GO and KEGG pathway enrichment analyses

Target genes of SGG against MDD were mapped into the Functional Annotation Tool DAVID 6.8 database (https://david.ncifcrf.gov/) for GO analysis, including the biological process (BP), cell component, and molecular function, and KEGG analysis. A heatmap was plotted by http://www.bioinformatics.com.cn, an online platform for data analysis and visualization.

## 3. Results

### 3.1. ADME properties of SGG

**Figure 1.**
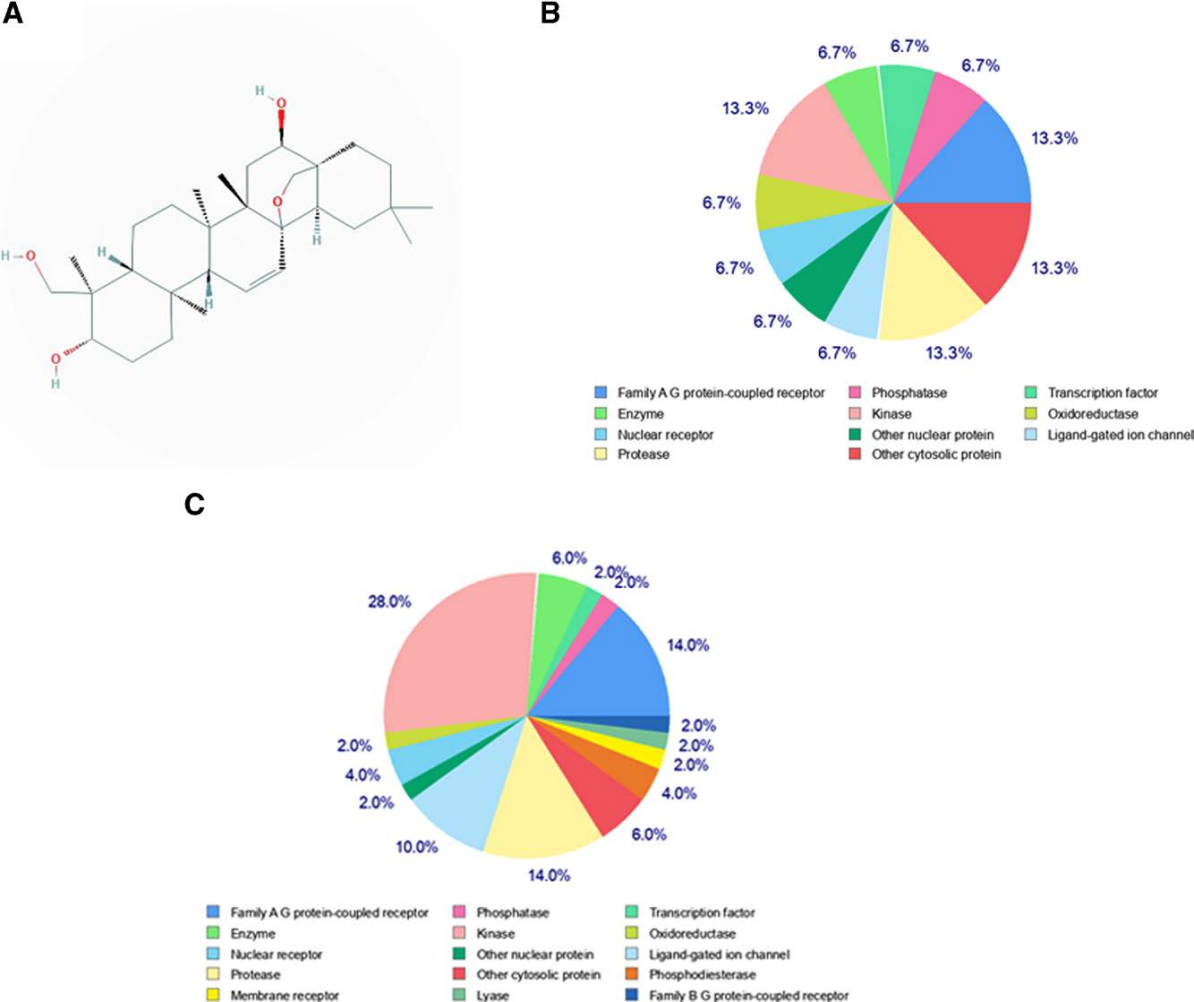
Prediction of the SGG target genes. (A) Chemical structure of SGG. (B) Classification of SGG target genes for top 15 according to its biochemical criteria. (C) Classification of SGG target genes for all according to its biochemical criteria. SGG = saikogenin G.

The chemical structure of SGG was obtained from the PubChem database and is shown in Figure 1A. In accordance with the RO5, SwissADME prediction showed that the molecular weight of SGG was 472.7 g/mol, which should be <500 g/mol, the topological polar surface area was 69.92 A^2^ < 140 A^2^, the octanol/water partition coefficient (Consensus LogP_0/W_) was 4.80 <5, the rotatable bond was 1 <10, the hydrogen bond donors was 3 no more than 5, and the hydrogen bond acceptors was 4 no more than 10.^[19]^ Results indicated that the properties of SGG were in line with the RO5, and GI absorption was high, suggesting that it had good drug-like properties (Table 1).

**Table 1:** Pharmacological and molecular properties of saikogenin G.

Property	Value
Molecular weight	472.7 g/mol
TPSA	69.92 A^2^
Consensus LogP_0/W_	4.80
Rotatable bonds	1
H-bond donor	3
H-bond acceptor	4
Molar refractivity	136.21
Log Kp (skin permeation)	−5.09 cm/s
Bioavailability score	0.55

TPSA = topological polar surface area.

### 3.2. Screening of targets of the SGG in MDD

Based on structure, potential targets of SGG were predicted via the SwissTargetPrediction and PharmMapper databases. In the SwissTargetPrediction database, targets with probability >0 were selected as potential targets (Fig. 1B, C). A total of 322 targets were obtained, including 27 duplicated targets (SwissTargetPrediction (Probability > 0) 67, PharmMapper 282).

**Figure 2.**
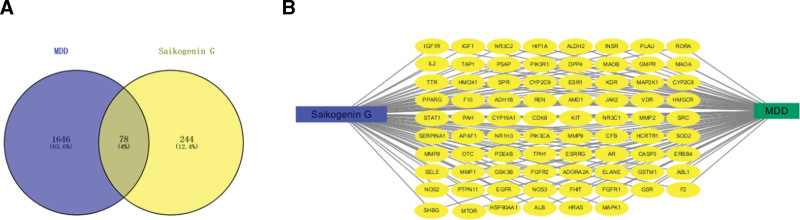
Targets of SGG against MDD. (A) Seventy-eight overlapping target genes between SGG and MDD. (B) Network of SGG, MDD, and all overlapping target genes. Yellow nodes represent the target genes. MDD = major depressive disorder, SGG = saikogenin G.

After removing redundant data, 1724 target genes relevant to MDD were obtained from the DisGeNET, DrugBank, Online Mendelian Inheritance in Man, and GeneCards databases. Then, a Venn diagram was used to outline 3 common targets in those databases. From an intersection of SGG compound targets and MDD targets, 78 overlapping genes were selected as targets of MDD treatment (Fig. 2).

### 3.3. PPI network analysis

**Figure 3.**
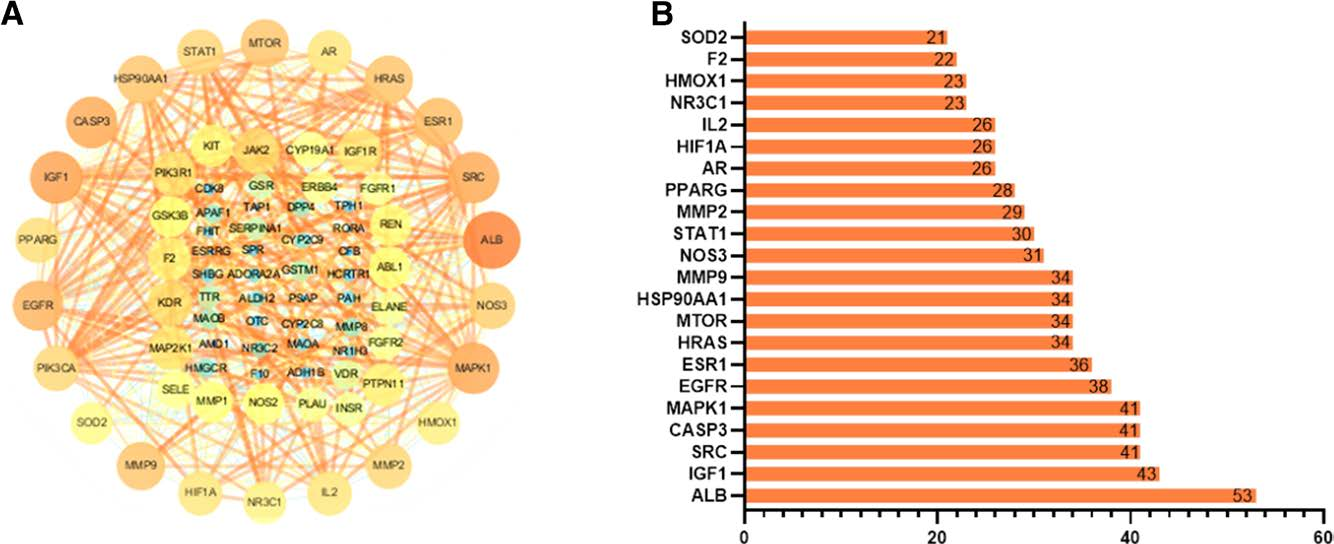
Interaction network of common targets. (A) Construction of a PPI network expressed by 76 common targets. Node size and color from orange (high) to wathet (low) represent the degree. The outer ring nodes represent the 22 big hub nodes. (B) Bar plot of the number of 22 hub node links. PPI = protein–protein interaction.

The PPI network of those 78 targets was set up with the STRING database. A total of 76 nodes and 646 edges were exhibited, and the average node degree was 16.6 with a medium confidence score of 0.400. The “Analysis network” tool in Cytoscape software was used to analyze the results and obtain the relevant parameters further (Fig. 3A). We selected key targets on the basis of the 3 parameters of “Degree,” “BetweennessCentrality,” and “ClosenessCentrality” above the median values of them. The threshold values were degree ≥ 20.5, ClosenessCentrality ≥ 0.547445, and betweenness ≥ 0.006381, and 22 hub nodes were obtained (Fig. 3B).

### 3.4. GO and KEGG pathway enrichment analysis

After using the DAVID 6.8 for enrichment analysis, we obtained 84 KEGG pathways and 296 GO biological processes (*P* < .05; Table 2). The first 10 results from the GO and KEGG pathway enrichment analyses were selected considering their *P* values.

**Table 2:** Information of 84 KEGG pathways.

No.	Term ID	Pathway name	*P* value	Count	Fold enrichment
1	hsa05205	Proteoglycans in cancer	6.28E–14	20	9.5542
2	hsa05200	Pathways in cancer	2.85E–12	24	5.8346
3	hsa05215	Prostate cancer	3.14E–12	14	15.1998
4	hsa04066	HIF-1 signaling pathway	1.76E–10	13	12.9379
5	hsa05230	Central carbon metabolism in cancer	6.94E–10	11	16.4212
6	hsa04915	Estrogen signaling pathway	3.91E–09	12	11.5808
7	hsa04151	PI3K-Akt signaling pathway	7.05E–09	19	5.2617
8	hsa04012	ErbB signaling pathway	1.52E–08	11	12.0800
9	hsa04015	Rap1 signaling pathway	2.05E–08	15	6.8244
10	hsa04917	Prolactin signaling pathway	3.54E–08	10	13.4566
11	hsa04014	Ras signaling pathway	5.21E–08	15	6.3413
12	hsa05219	Bladder cancer	1.57E–07	8	18.6423
13	hsa04919	Thyroid hormone signaling pathway	2.26E–07	11	9.1388
14	hsa05214	Glioma	2.72E–07	9	13.2289
15	hsa05218	Melanoma	5.46E–07	9	12.1109
16	hsa04550	Signaling pathways regulating pluripotency of stem cells	1.42E–06	11	7.5068
17	hsa04370	VEGF signaling pathway	2.59E–06	8	12.5301
18	hsa04068	FoxO signaling pathway	8.27E–06	10	7.1300
19	hsa05213	Endometrial cancer	1.37E–05	7	12.8614
20	hsa05161	Hepatitis B	1.57E–05	10	6.5891
21	hsa04726	Serotonergic synapse	1.63E–05	9	7.7466
22	hsa05223	Nonsmall cell lung cancer	2.11E–05	7	11.9427
23	hsa05221	Acute myeloid leukemia	2.11E–05	7	11.9427
24	hsa04510	Focal adhesion	4.37E–05	11	5.1017
25	hsa04960	Aldosterone-regulated sodium reabsorption	4.45E–05	6	14.6987
26	hsa05211	Renal cell carcinoma	5.44E–05	7	10.1332
27	hsa05231	Choline metabolism in cancer	7.35E–05	8	7.5677
28	hsa05220	Chronic myeloid leukemia	8.91E–05	7	9.2888
29	hsa04931	Insulin resistance	1.13E–04	8	7.0772
30	hsa00330	Arginine and proline metabolism	1.50E–04	6	11.465
31	hsa04722	Neurotrophin signaling pathway	2.18E–04	8	6.3694
32	hsa04152	AMPK signaling pathway	2.54E–04	8	6.2141
33	hsa04914	Progesterone-mediated oocyte maturation	2.55E–04	7	7.6873
34	hsa04810	Regulation of actin cytoskeleton	2.79E–04	10	4.5496
35	hsa05160	Hepatitis C	4.10E–04	8	5.7469
36	hsa05210	Colorectal cancer	4.16E–04	6	9.2460
37	hsa04910	Insulin signaling pathway	5.12E–04	8	5.5386
38	hsa05212	Pancreatic cancer	5.19E–04	6	8.8192
39	hsa04660	T-cell receptor signaling pathway	5.42E–04	7	6.6879
40	hsa04062	Chemokine signaling pathway	6.09E–04	9	4.6230
41	hsa00982	Drug metabolism – cytochrome P450	6.39E–04	6	8.4301
42	hsa04662	B-cell receptor signaling pathway	6.84E–04	6	8.3080
43	hsa04668	TNF signaling pathway	7.76E–04	7	6.2504
44	hsa04520	Adherens junction	7.80E–04	6	8.0739
45	hsa04930	Type II diabetes mellitus	1.44E–03	5	9.9523
46	hsa04650	Natural killer cell–mediated cytotoxicity	1.54E–03	7	5.4819
47	hsa05152	Tuberculosis	2.20E–03	8	4.3183
48	hsa04912	GnRH signaling pathway	2.38E–03	6	6.2995
49	hsa04150	mTOR signaling pathway	2.91E–03	5	8.2364
50	hsa05216	Thyroid cancer	3.18E–03	4	13.1782
51	hsa04730	Long-term depression	3.29E–03	5	7.9618
52	hsa04664	Fc epsilon RI signaling pathway	5.16E–03	5	7.0251
53	hsa04610	Complement and coagulation cascades	5.44E–03	5	6.9233
54	hsa04725	Cholinergic synapse	5.59E–03	6	5.1644
55	hsa04071	Sphingolipid signaling pathway	7.74E–03	6	4.7771
56	hsa00380	Tryptophan metabolism	7.92E–03	4	9.5542
57	hsa05164	Influenza A	8.84E–03	7	3.8436
58	hsa05206	MicroRNAs in cancer	8.85E–03	9	3.0066
59	hsa04380	Osteoclast differentiation	1.11E–02	6	4.3760
60	hsa05222	Small cell lung cancer	1.13E–02	5	5.6201
61	hsa05162	Measles	1.18E–02	6	4.31015
62	hsa04540	Gap junction	1.27E–02	5	5.428504
63	hsa00360	Phenylalanine metabolism	1.29E–02	3	16.86029
64	hsa04913	Ovarian steroidogenesis	1.38E–02	4	7.79932
65	hsa04630	Jak-STAT signaling pathway	1.66E–02	6	3.953448
66	hsa00220	Arginine biosynthesis	1.77E–02	3	14.33125
67	hsa04921	Oxytocin signaling pathway	1.90E–02	6	3.821667
68	hsa04916	Melanogenesis	1.94E–02	5	4.777083
69	hsa04932	Nonalcoholic fatty liver disease	.0195	6	3.796358
70	hsa00340	Histidine metabolism	.0212	3	13.02841
71	hsa05142	Chagas disease (American trypanosomiasis)	.0221	5	4.593349
72	hsa04620	Toll-like receptor signaling pathway	.0235	5	4.506682
73	hsa04210	Apoptosis	.0258	4	6.163978
74	hsa05145	Toxoplasmosis	.0265	5	4.342803
75	hsa04114	Oocyte meiosis	.0273	5	4.303679
76	hsa05202	Transcriptional misregulation in cancer	.0286	6	3.432635
77	hsa04670	Leukocyte transendothelial migration	.0306	5	4.153986
78	hsa04320	Dorsoventral axis formation	.0312	3	10.61574
79	hsa05120	Epithelial cell signaling in *Helicobacter pylori* infection	.0315	4	5.70398
80	hsa05034	Alcoholism	.0355	6	3.238701
81	hsa05140	Leishmaniasis	.0366	4	5.382629
82	hsa04611	Platelet activation	.0448	5	3.674679
83	hsa04010	MAPK signaling pathway	.0457	7	2.643445
84	hsa05204	Chemical carcinogenesis	.0493	4	4.777083

AMPK = adenosine 5‘-monophosphate (AMP)-activated protein kinase, FoxO = forkhead box O, GnRH = gonadotropin-releasing hormone, KEGG = Kyoto Encyclopedia of Genes and Genomes, MAPK = mitogen-activated protein kinase, mTOR = mammalian target of rapamycin, VEGF = vascular endothelial growth factor.

**Figure 4.**
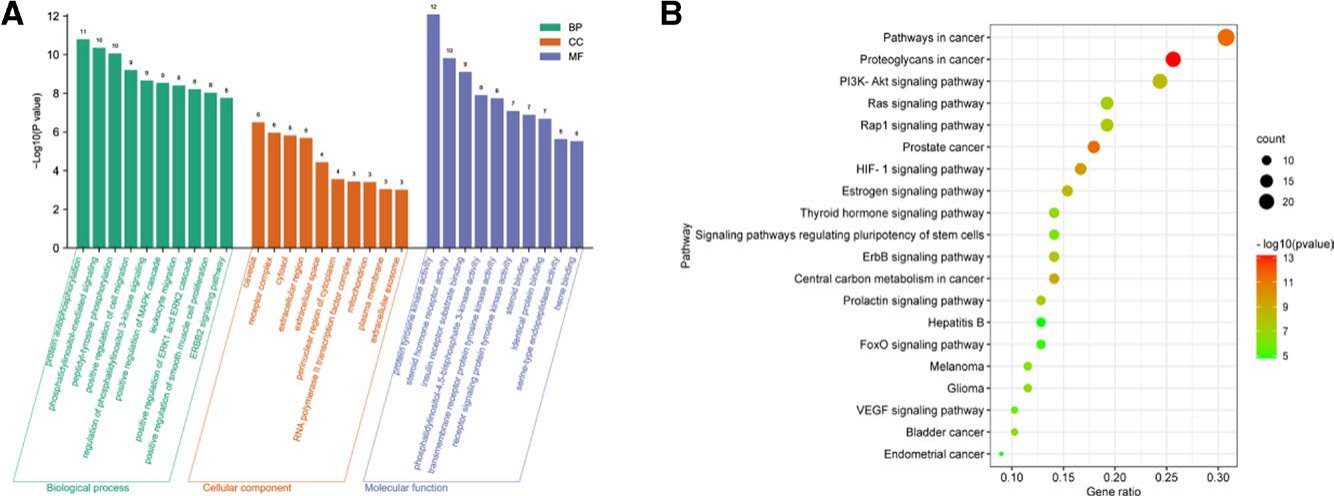
GO and KEGG enrichment analysis of the targets. (A) Top 10 significantly enriched terms in the GO biological processes, cell component, and molecular function. (B) Bubble chart of the top 20 significantly enriched terms in KEGG pathways. GO = gene ontology, KEGG = Kyoto Encyclopedia of Genes and Genomes.

As shown in Figure 4A, the top 10 significantly enriched BP terms were protein autophosphorylation, phosphatidylinositol-mediated signaling, peptidyl-tyrosine phosphorylation, positive regulation of cell migration, regulation of phosphatidylinositol 3-kinase signaling, positive regulation of mitogen-activated protein kinase cascade, leukocyte migration, positive regulation of extracellular signal-regulated kinase (ERK)1 and ERK2 cascade, positive regulation of smooth muscle cell proliferation, and ERBB2 signaling pathway.

The top 10 enriched cell component terms were caveola, receptor complex, cytosol, extracellular region, extracellular space, perinuclear region of cytoplasm, RNA polymerase II transcription factor complex, mitochondrion, plasma membrane, and extracellular exosome. The top 10 enriched molecular function terms included protein tyrosine kinase activity, steroid hormone receptor activity, insulin receptor substrate binding, phosphatidylinositol-4,5-bisphosphate 3-kinase activity, transmembrane receptor protein tyrosine kinase activity, receptor signaling protein tyrosine kinase activity, steroid binding, identical protein binding, serine-type endopeptidase activity, and heme binding.

Ranking by *P* value, the top 20 pathways are shown in Figure 4B. The first 10 signaling pathways were proteoglycans in cancer (hsa05205), pathways in cancer (hsa05200), prostate cancer (hsa05215), hypoxia-inducible factor-1 (HIF-1; hsa04066), central carbon metabolism in cancer (hsa05230), estrogen (hsa04915), PI3K-Akt (hsa04151), ErbB (hsa04012), Rap1 (hsa04015), and prolactin (hsa04917).

## 4. Discussion

Major depressive disorder is a multifactorial mental disorder caused by the interplay between environmental factors and genetic susceptibility. ^[20]^ Disorders in heredity, neurological hormone, immune function, bioenergetics, mitochondrial oxidative stress, vasomotor factors and free radicals, gender, and developmental factors are associated with MDD.^[21–23]^ Existing drugs are not completely effective because of the complex etiology of MDD. Traditional Chinese Medicine has a long history and few side effects in the treatment of MDD. Thus, finding new drugs from Chinese herbal medicine is important.

Gene ontology enrichment analysis showed the complex and comprehensive effects of SGG and revealed several BP terms, such as autophosphorylation, phosphorylation, ERK1 and ERK2 cascade, and mitogen-activated protein kinase cascade. KEGG pathway analysis was performed to reveal the underlying mechanism of antidepressive with SGG, including the HIF-1, estrogen, PI3K-Akt, and ErbB signaling pathways.

Hypoxia-inducible factor-1 is an important transcriptional factor responding to hypoxia, a heterodimer composed of HIF-1α (regulatory subunit) and HIF-1β (constitutive subunit). HIF-1 signaling pathways regulate various BPs, including aging, proliferation and apoptosis, anaerobic metabolism, TCA cycle metabolism, oxygen consumption, inflammation, and angiogenesis.^[24,25]^ Previous studies suggested that a close functional interaction exists between HIF-1 and glucocorticoid receptor (major part of stress response).^[26]^ Li et al^[27]^ found that inhibitor of prolyl hydroxylase (FG-4592) reverses depression-like behavior through the activation of the HIF-1 signaling pathway. Kang et al^[22]^ assumed that elevated levels of HIF-1 activate the phosphocreatine-creatine kinase circuits and regularize the brain energy metabolism as a result of antidepressants via normalization of structural and functional alterations. Insulin, interleukin (IL)-1β, and epidermal growth factor induce the expression of HIF-1α by activating PI3K-Akt signaling pathways. In addition, a previous report revealed that the overexpression of HIF-1α upregulates the expression of inflammatory factors (IL-13, IL-9, IL-1, and tumor necrosis factor-α) by activating the epidermal growth factor receptor/PI3K/AKT pathway.^[28]^

PI3K-Akt signaling plays an important role in many diseases, including cancer, diabetes, obesity, cardiovascular diseases, and neurological diseases (depression, anxiety).^[29–31]^ Cao et al^[23]^ reported that icariin significantly ameliorates the seeming symptoms in rats with perimenopausal depression depending on the regulation of PI3K-Akt signaling. Insulin-like growth factor 1, creatine, schisandrin, and liquiritigenin exert an antidepressant effect on animals with depression induced by chronic stress or corticosterone through the PI3K-Akt pathway.^[29–32]^

Akt (a target of PI3K) acts as a pivot position in the treatment of depression through connecting with downstream targets, including glycogen synthase kinase-3 beta (GSK3β), mammalian target of rapamycin, IκB kinaseα, γ-aminobutyric acid A receptor, and forkhead box O, which are involved in the regulation of glucose and lipid metabolism, synaptic plasticity, angiogenesis, proliferation and apoptosis, synaptic signaling, and mitochondrial function. Moreover, PI3K and AKT activate immune cells by regulating key inflammatory cytokines (IL-8, IL-10, IL-12, IL-23, interferon-α, and interferon-β) and are related to the action of dopamine and serotonin.^[33,34]^ Depression has been associated with increased activity of the immune system and the release of proinflammatory cytokines (IL-1β, IL-6, and tumor necrosis factor-α), which are associated with hypothalamic–pituitary–adrenal axis abnormalities in clinical and translational studies.^[35,36]^ Moreover, recent studies have focused on neuroimmune processes because they may involve the pathophysiology of MDD directly and indirectly through some neurobiological processes related to neuroplasticity-related systems, monoamine system, and the hypothalamic–pituitary–adrenal axis.^[37]^

GSK3β as a downstream target of AKT is crucial for the function of the central nervous system in humans. Phosphorylation on Tyr216 and Ser9 of GSK3β represents positive and negative activities, respectively.^[38]^ Extracellular factors serve many functions, such as regulating the PI3K/AKT pathway and then inhibiting GSK3β by phosphorylation of Ser9.^[38]^ Studies using animal models have indicated that inhibiting the activation of Akt-GSK3β signaling impairs the survival and synaptic plasticity of hippocampal neurons and induces depressive behavior.^[39]^ Further study found that resveratrol may show antidepressant effect by activating the Akt/GSK3β signaling pathway.^[35]^ Phosphatase-mediated phosphorylation plays an important role in the processes of most signaling pathways mentioned above. These situations are consistent with our results that the enriched BP terms are protein autophosphorylation, phosphatidylinositol-mediated signaling, and peptidyl-tyrosine phosphorylation.

The deficiency of central monoamine neurotransmitters dopamine (DA) and serotonin is closely related to depression. At present, selective serotonin reuptake inhibitors, such as fluoxetine, paroxetine, sertraline, fluvoxamine, and citalopram, are widely used in the treatment of depression. Previous studies have shown that the ErbB signaling pathway plays a critical role in depression.^[40–42]^ Four closely related ErbB subtypes, namely, ErbB1, ErbB2, ErbB3, and ErbB4, have been identified in humans. Several researchers have reported that NRG1/ErbB signaling plays an important role in the modulation of the midbrain DA system.^[40,43–47]^ A recent study has confirmed that ketamine exhibits antidepressant effects in adult mice depending on NRG1/ErbB4 signaling.^[41]^ In addition, ErbBs can activate the PI3K pathway directly or indirectly.

Tadeus Reichstein, Adolf Butenandt, and Edward Adelbert Doisy were the first to isolate and characterize estrogen in 1929.^[48]^ Estrogen, including estrone, estradiol, estriol, and estetrol, is a steroid hormone. Estrogen is involved in various physiological processes, including breast and sexual organ development, menstrual cycle, reproduction, cardiovascular protection, bone integrity, cognitive, neuroprotective effects, and antiinflammatory effects, by interacting with estrogen receptors.^[39,49]^ Women show a higher incidence and prevalence of MDD than men. The occurrence of depression increases during the menopause and postpartum periods when the level of estrogen decreases significantly. Several studies have demonstrated that estrogen performs favorable effects in MDD via modulating monoamine neurotransmitters (DA and 5-hydroxytryptamine), inflammatory processes, and dendritic spine number.^[50,51]^ The activation of ERK and PI3K elevates after acute treatment with 17β-estradiol. Estrogen is an important adjunct to antidepressant drug treatments clinically.

**Figure 5.**
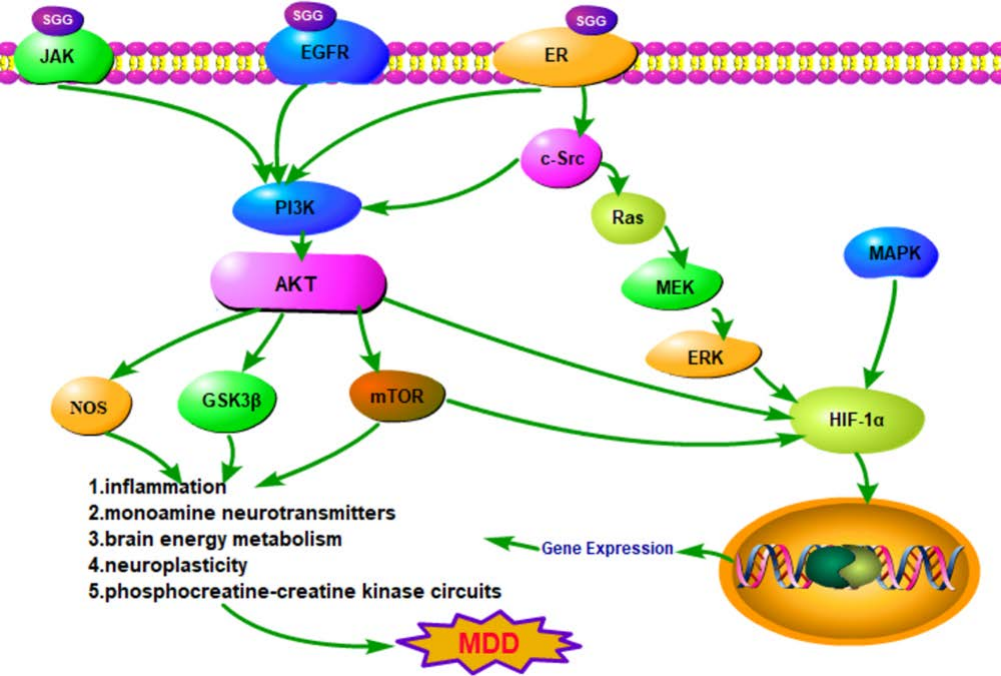
Schematic diagram of crucial pathways and primary targets of SGG in the treatment of MDD. MDD = major depressive disorder, SGG = saikogenin G.

The HIF-1, Estrogen, PI3K-Akt, and ErbB signaling pathways are interrelated and interact with each other. We identified key proteins targeted by SGG in the pathways and GO enrichment above and show the related biomechanism in Figure 5.

## 5. Conclusion

This study elucidated the potential mechanisms of SGG in treating MDD. Bioinformatics analysis showed that SGG exhibits good drug-like properties, with 78 genes selected as targets of SGG in MDD treatment. Our study also suggested that HIF-1, estrogen, PI3K-Akt, and ErbB signaling pathways regulate inflammation, energy metabolism, dendritic spine number, phosphocreatine-creatine kinase circuits, and BPs in the treatment. This study provided valuable information to investigate the comprehensive and underground effect of SGG in MDD treatment.

## Author contributions

**Conceptualization:** Lili Hu, Jue Wang, Xiaoge Zhao.

**Data curation:** Lili Hu.

**Formal analysis:** Lili Hu, Jue Wang, Donghui Cai.

**Funding acquisition:** Lili Hu, Jue Wang.

**Investigation:** Lili Hu, Xiaoge Zhao.

**Methodology:** Lili Hu, Jue Wang, Donghui Cai.

**Project administration:** Lili Hu, Donghui Cai.

**Resources:** Lili Hu, Jue Wang, Donghui Cai.

**Software:** Lili Hu.

**Supervision:** Lili Hu.

**Validation:** Lili Hu, Xiaoge Zhao.

**Visualization:** Lili Hu, Xiaoge Zhao.

**Writing – original draft:** Lili Hu.

**Writing – review & editing:** Lili Hu, Donghui Cai.
